# Malnutrition-related hyperammonemic encephalopathy presenting with burst suppression: a case report

**DOI:** 10.1186/s13256-019-2185-6

**Published:** 2019-08-10

**Authors:** Antonio Leidi, Marisa Pisaturo, Thierry Fumeaux

**Affiliations:** 1 Department of Internal Medicine, GHOL-Nyon Hospital, Chemin Monastier 10, 1260 Nyon, Switzerland; 2 Department of Internal Medicine and Critical Care Medicine, GHOL-Nyon Hospital, Chemin Monastier 10, 1260 Nyon, Switzerland

**Keywords:** Hyperammonemia, Coma, Malnutrition, Burst suppression, EEG

## Abstract

**Background:**

Hyperammonemia is a common cause of metabolic encephalopathy, mainly related to hepatic cirrhosis. Numerous nonhepatic etiologies exist but they are infrequent and not well known, thus, leading to misdiagnosis and inadequate care. Electroencephalography has a proven diagnostic and prognostic role in comatose patients. Burst suppression is a preterminal pattern found in deep coma states and is rarely associated with metabolic causes.

**Case presentation:**

We report the case of an 81-year-old Caucasian man presenting with rapidly progressive somnolence and mutism. Soon after his arrival in our hospital, he developed profound coma. A comprehensive diagnostic workup was unremarkable except for admission electroencephalography showing diffuse slowing of cerebral activity with an intermittent pattern of burst suppression. He was admitted to our intensive care unit for supportive care where malnutrition-related hyperammonemia was diagnosed. His clinical course was spontaneously favorable and follow-up electroencephalography demonstrated normal cerebral activity.

**Conclusions:**

Nonhepatic hyperammonemia is a rare and potentially reversible cause of encephalopathy. Ammonia level measurement should be part of the diagnostic workup in patients with unexplained coma, particularly in the setting of nutritional deficiencies or nutritional supply. Detection of diffuse and nonspecific mild to moderate slowing of cerebral activity (theta-delta ranges) on electroencephalography is common. In contrast, to the best of our knowledge, burst suppression has never been described in association with hyperammonemia.

## Background

Ammonia (NH3+) and its associated acid ammonium (NH4+) result from the catabolism of amino acids (AA). For simplicity, in this case report the term ammonia refers to both forms. In healthy individuals, the main source of ammonia is digestive from intestinal bacterial degradation of proteins and urea. Catabolism of AA continually produces ammonia in human organs. The kidney is another main source, which produces ammonia in the proximal tubular cells in order to buffer H+ in urine. Elimination of ammonia in a healthy individual takes place in the liver, where it is transformed into urea by periportal hepatocytes (the urea cycle). Urea, a nontoxic molecule, is subsequently eliminated in the urine and colon. A second key actor of ammonia metabolism is glutamine synthase (GS), which produces glutamine by incorporating ammonia in glutamate. This enzyme is present in periportal hepatocytes and prevents the release of residual ammonia into the systemic circulation. Produced glutamine is then transported to the small intestine, where ammonia is released, and then carried back to the liver via the portal system. GS is also expressed in muscles, kidneys, and astrocytes acting as an initial adaptive mechanism in case of hyperammonemia. Finally, the kidney can increase ammonia excretion in urine from 30% up to 70% [1–3]. Ammonia gets access to the brain through passive diffusion and mediated transport. It is then combined with glutamate by astrocytes to produce glutamine, which is provided to neurons, reversed back to glutamate and then released as a neurotransmitter. When present in an excessive amount, ammonia has a selective neurotoxicity. Clinical manifestations range from irritability to seizure, coma, cerebral herniation, and death [2]. Hosting the urea cycle, the liver plays an essential role in ammonia detoxification; thus, up to 90% of cases of hyperammonemia are related to hepatic diseases. Apart from hepatic dysfunction, two mechanisms may lead to ammonia excess: increased production and decreased metabolism (Table 1). Ammonia production is raised in cases of exaggerated protein supply (enteral or parenteral), muscle protein catabolism (seizure, trauma, malnutrition), urinary tract infection with urease producing bacteria, and hemato-oncological disorders. Ammonia elimination is altered in cases of inborn errors of metabolism, by medication interfering with urea cycle (for example, valproate), and by portosystemic shunting. Constitutional defects of ammonia metabolism may only be apparent in adulthood, following a precipitating event [3].

**Table 1:** Mechanism leading to nonhepatic hyperammonemia: increased production and decreased metabolism

Overproduction	Undermetabolism
Excessive protein supply (enteral or parenteral)	Portosystemic shunting
Muscles catabolism: cachexia, starvation, seizure	Medications interfering with urea cycle: valproic acid, carbamazepine, salicylate, glycine, ribavirin
Urease producing bacteria urinary tract infections: *Proteus mirabilis, Klebsiella* species, *Escherichia coli, Morganella morganii, Providencia rettgeri,* diphtheroids	Inborn error of metabolism: inherited defects of the urea cycle, amino acid transporters, fatty acid oxidation, organic acid disorders
Hematological malignancies: multiple myeloma, leukemia	

Electroencephalography (EEG) has a well-recognized role in the evaluation of altered consciousness states. It is a diagnostic and monitoring tool, providing prognostic information. With worsening of metabolic encephalopathies, gradual EEG progression is observed, similar to changes described in drug-induced coma. As the amplitude increases, the dominant EEG frequency declines and the length of flat periods increases toward a completely isoelectric trace [4]. The burst suppression pattern consists of alternative periods of slow waves with high amplitude (the burst phase) and periods of marked activity depression (the suppression phase). It is associated with profound coma of various etiologies (cerebral anoxia, hypothermia, anesthesia, drugs intoxications) [5]. To the best of our knowledge, we report here the first case of an adult with hyperammonemic coma presenting with burst suppression on admission EEG.

## Case presentation

**Fig. 1:**
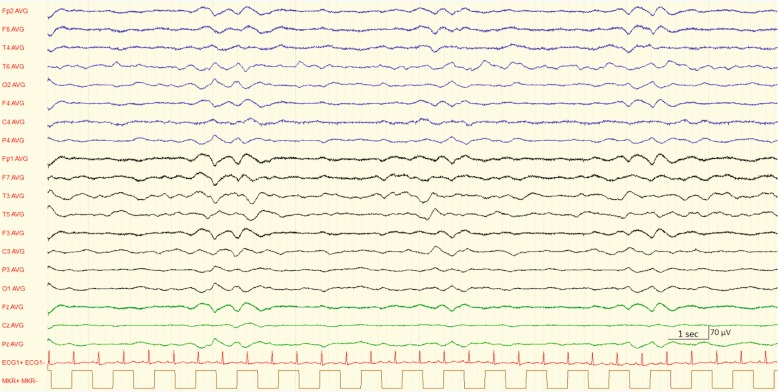
Electroencephalography at admission with burst suppression pattern: periods of slow waves (the burst) alternate with period of electroencephalography activity suppression (LoFil 0.53 Hz, Hifil 70 Hz)

An 81-year-old Caucasian man known only for benign prostatic hyperplasia was referred for loss of appetite and poor food intake, with rapidly progressing somnolence and mutism. On admission at our emergency department, he developed a deep coma state. A physical examination revealed an alteration of consciousness (Glasgow Coma Scale 4/15) without any other relevant signs. Routine laboratory values were in the normal range. A urine culture showed no bacterial growth and toxicology screening was negative. A head computed tomography (CT) scan was normal and cerebrospinal fluid analysis showed mildly raised proteins (664 mg/l), with no white cells. Non-convulsive status epilepticus was suspected, and he was treated with a loading dose of levetiracetam (1600 mg intravenously administered) before being admitted to our intensive care unit (ICU). The EEG performed on admission (Fig. 1) showed a diffuse slow activity with an intermittent burst suppression pattern. In the absence of any other evident cause for coma, his ammonium level was measured and proved to be high (259 μmol/l, normal value < 60), with subsequent confirmation. Supportive care was initiated, and evolution at 48 hours showed complete clinical recovery and normalization of blood ammonia level. A follow-up EEG demonstrated normal cerebral activity. The etiological assessment following clinical recovery revealed low blood levels of several essentials AA pointing to malnutrition-related hyperammonemia. He was satisfied with the care he received.

## Discussion and conclusions

We describe the case of a patient presenting with a rapidly progressive coma state and an unusual burst suppression EEG pattern. An initial diagnostic workup failed to reveal any evident etiology, including hepatic dysfunction. Dosing of ammonia level allowed us to diagnose nonhepatic hyperammonemic encephalopathy (NHHE). Although this rare condition is usually associated with a poor prognosis [6], our patient’s clinical course with supportive care was marked by complete clinical recovery. This case highlights the necessity of including ammonia level measurement in the diagnostic workup of unexplained coma, even in the absence of hepatic dysfunction. Early diagnosis is important as it may be lethal if unrecognized and untreated. Severe hepatic hyperammonemic encephalopathy and NHHE usually induce a nonspecific diffuse symmetric slowing of electrographic cerebral activity ranging from theta to delta waves. The only report we found of burst suppression was in a 13-year-old girl with high-dose (> 50 mg/kg) valproic acid-induced hyperammonemic encephalopathy. However, causal association was not assessable due to many confounding factors, particularly the administration of high-dose and combined anti-epileptic drugs (midazolam, lorazepam, fosphenytoin, and phenobarbital) before EEG was performed [7]. We therefore believe that our case describes the first association between such an EEG pattern and hyperammonemic encephalopathy, moreover, with spontaneous complete recovery. Although probably rare, this combination is certainly underreported, either because measurement of ammonia levels is not routinely performed, or because EEG and ammonia measurement are usually not done simultaneously.

## Data Availability

All data generated or analyzed during this study are included in this published article.

## Abbreviations

AA: Amino acids
EEG: Electroencephalography
GS: Glutamine synthase
NHHE: Nonhepatic hyperammonemic encephalopathy
